# Lack of association of *miR-146a* and *Ets-1* gene polymorphisms with Fuchs uveitis syndrome in Chinese Han patients

**Published:** 2012-02-11

**Authors:** Qingyun Zhou, Aize Kijlstra, Shengping Hou, Hongsong Yu, Xuedong Zhang, Xinyu Li, Xiang Xiao, Peizeng Yang

**Affiliations:** 1 The First Affiliated Hospital of Chongqing Medical University, Chongqing Key Laboratory of Ophthalmology, and Chongqing Eye Institute, Chongqing, P.R. China; 2 Eye Research Institute Maastricht, Department of Ophthalmology, University Hospital Maastricht, Maastricht, the Netherlands

## Abstract

**Purpose:**

The aim of this study was to investigate the potential association of microRNA-146a (*miR-146a*) and V-Ets oncogene homolog 1 (*Ets-1*) gene polymorphisms with Fuchs Uveitis syndrome (FUS).

**Methods:**

Three single-nucleotide polymorphisms (SNPs), *miR-146a*/rs2910164, *ets-1*/rs1128334, and *ets-1*/rs10893872 were genotyped in 219 Han Chinese patients with FUS and 612 healthy controls using the polymerase chain reaction-restriction fragment length polymorphism (PCR-RFLP) method. Genotype counts were analyzed by the χ^2^ test.

**Results:**

No significant difference concerning the genotypic and allelic frequencies of rs2910164, rs1128334, and rs10893872 polymorphisms could be found between patients with FUS and the normal controls. Analysis according to gender did not show any influence of sex on the association of *miR-146a* and *Ets-1* with FUS.

**Conclusions:**

Our results suggest that the investigated three SNPs, *miR-146a*/rs2910164, *ets-1*/rs1128334, and *ets-1*/rs10893872, are not associated with FUS in the Han Chinese population.

## Introduction

Fuchs Uveitis Syndrome (FUS) is a chronic inflammatory eye disease, which usually presents as unilateral anterior uveitis in young adults [1]. It is a speciﬁc uveitis entity usually manifesting as a mild unilateral anterior uveitis with stellate medium-sized keratic precipitates (KP) and varying degrees of iris depigmentation or heterochromia [2-4]. In Chinese patients it occurs mostly in the third or fourth decades of life and affects both genders equally. Complicated cataract and secondary glaucoma are common in these patients [3]. Although the etiology of FUS has not yet been fully understood, several studies have revealed that both viral infection and genetic risk factors may be involved in its pathogenesis [5-7]. Makley et al. [8] found that monozygotic twins both developed Fuchs’ syndrome. Earlier reports have found that several human leukocyte antigen (HLA) genes, such as *HLA-DR_W_53* and *HLA-C_W_3* were associated with FUS [7]. Furthermore, recent studies have shown the association of several non-HLA genes including the cytotoxic T cell antigen (*CTLA*) *4* gene, the intercellular adhesion molecule 1 (*ICAM-1*) gene and the interleukin 23 receptor (*IL23R*) gene with this disease [9-11].

As an abundant class of highly conserved small noncoding RNAs (18–25 nucleotides long), miRNAs play critical roles in the regulation of diverse biologic processes, such as development, infection, immune response, inflammation and tumorigenesis [12,13]. microRNA-146a (*miR-146a*) was reported as a negative regulator of innate immunity in systemic lupus erythematosus (SLE) patients [14] and was indicated to be the key regulator during viral infection [15-17]. Recent studies have demonstrated that single nucleotide polymorphisms (SNPs) located either in the pre-miRNAs or within miRNA-binding sites are likely to affect the expression of the miRNA targets and, thus, may contribute to the susceptibility to disease [18,19]. V-Ets oncogene homolog 1 (Ets-1) is the first member of the Ets oncogene family which contributes to tumor development and progression [20]. It has recently been identified to bind to the *miR-146a* promoter region and the Ets-1 level was shown to markedly affect *miR-146a* promoter activity in vitro and the knockdown of Ets-1 directly impaired the induction of *miR-146a* [21]. SNPs rs1128334 and rs10893872 located in the 3′UTR of *Ets-1* are on putative miRNA binding sites and are both associated with SLE in Asian populations [22].

In view of the important role of *miR-146a* in the development of innate immunity and virus infection associated diseases and the potential modulatory effect of *Ets-1* on *miR-146a* expression, we investigated the association of rs2910164, rs1128334, and rs10893872 of both genes with FUS. Unfortunately, we failed to find any association of the aforementioned three polymorphisms with this disease in a Han Chinese population.

## Methods

### Study populations

The study groups comprised 219 unselected, consecutive Han Chinese patients with FUS and 612 age-, sex-, ethnic-matched healthy controls who were recruited from the Uveitis Study Center of the Sun Yat-sen University (Guangzhou, P.R. China) and the First Affiliated Hospital of Chongqing Medical University (Chongqing, P.R. China). The diagnosis of FUS was principally based on clinical manifestations as described earlier [3]. The study was approved by the Local Ethics Research Committee of The First Affiliated Hospital of Chongqing Medical University, and informed consent was obtained from all tested subjects. The tenets of the Declaration of Helsinki were conducted during all procedures of this study.

### Genomic DNA extraction and genotyping

Genomic DNA samples of patients with FUS and healthy controls were extracted by using the QIAamp DNA Blood Mini Kit (Qiagen, Hilden, Germany). Amplification of the target DNA sequence in the *miR-146a* and *Ets-1* gene was analyzed by the polymerase chain reaction (PCR) using primers presented in Table 1. Each PCR reaction was conducted in a 10 μl reaction volume containing 5 μl Premix Taq (Ex Taq Version; TaKaRa Biotechnology Co. Ltd., Dalian, China), 20 pmol primers, and 0.2 μg of genomic DNA for amplification of the DNA. The tested three SNPs were genotyped by PCR-restriction fragment length polymorphism (RFLP) analysis. PCR products were digested with 2 U of restriction enzymes HSP92II at 37 °C (Promega, Madison, WI) and TSP509I at 65 °C (Fermentas, Shenzhen, China) overnight. PCR fragments were separated on 4% agarose gels. Twenty percent of the PCR samples were directly sequenced to confirm the polymerase chain restriction fragment length polymorphism (PCR-RFLP) results (Invitrogen Biotechnology Co., Guangzhou, China).

**Table 1:** Primers and restriction enzymes used in RFLP analysis.

**SNP**	**Primers**	**Tm (°C)**	**Restriction enzymes**
rs2910164	5′-ATGGGTTGTGTCAGTGTCAGACAT-3′	58	HSP92II
	5′-TGCCTTCTGTCTCCAGTCTTCCAA-3′		
rs1128334	5′-TATTGTGTTTGACTATTTTCCAACAT-3′	55	HSP92II
	5′-ACTTACATCGCTACATCTCT-3′		
rs10893872	5′-ATCCCAGACCAAACCCAGTA-3′	60	TSP509I
	5′-TGGGCAGTAACAGGCTCTTT-3′		

RFLP, restriction fragment length polymorphism.

### Statistical analysis

Hardy–Weinberg equilibrium (HWE) was tested in the subjects using the χ^2^ test. Genotype and allele frequencies were compared between patients and controls by the χ^2^ test using SPSS version 17.0 (SPSS, Inc., Chicago, IL). Bonferroni correction was used to account for multiple testing. A two-tailed pP value <0.05 was considered to be statistically significant.

## Results

Two hundred and nineteen consecutive Han Chinese patients with FUS and 612 healthy controls were enrolled in the present study. The average age of the patients with FUS and normal controls were 36.4±12.4 years (range: 16 to 62 years) and 34.6±11.9 years (range: 18 to 57 years) in the present study, respectively. The age and gender distribution of the patients with FUS and controls are shown in Table 2.

**Table 2:** Age and gender distribution in Fuchs’ patients and controls.

**Group**	**Age (y; mean±SD)**	**Age range**	**Male (%)**	**Female (%)**
Controls	34.6±11.9	18–57	350 (57.2%)	262 (42.8%)
Fuchs’ patients	36.4±12.4	16–62	117 (53.4%)	102 (46.6%)

Three SNPs (rs2910164, rs1128334, and rs10893872) were successfully genotyped in 219 patients with FUS and 612 healthy controls. The results showed that the distribution of the genotype and the alleles for each SNP did not deviate from the Hardy–Weinberg equilibrium (HWE) in FUS and healthy controls (p>0.05). The distribution of genotypic and allelic frequencies of the three tested polymorphisms is presented in Table 3. There was no statistically significant difference concerning the genotype and allele of these three SNPs rs2910164, rs1128334, and rs10893872 between Fuchs’ patients and controls. In addition, we failed to find any influence of sex on the association of the tested gene polymorphisms with FUS by stratification analysis according to gender.

**Table 3:** Frequencies of genotypes and alleles of *miR-146a* and *Ets-1* polymorphism in Fuchs’ patients and controls.

**SNP**	**Genotype allele**	**FUS (n=219)**	**Controls (n=612)**	**χ^2^**	**p value**	**pc value**	**OR (95% CI)**
rs2910164	GG	36 (0.164)	79 (0.129)	1.69	0.21	NS	1.33 (0.87–2.04)
	GC	91 (0.416)	279 (0.456)	1.06	0.34	NS	0.85 (0.62–1.16)
	CC	92 (0.42)	254 (0.415)	0.02	0.94	NS	1.02 (0.75–1.40)
	G	514 (0.431)	437 (0.357)	0.32	0.6	NS	1.07 (0.85–1.34)
	C	678 (0.569)	787 (0.643)	0.32	0.6	NS	0.94 (0.75–1.18)
rs1128334	AA	30 (0.137)	72 (0.118)	0.56	0.47	NS	1.19 (0.75–1.88)
	AG	85 (0.388)	276 (0.451)	2.59	0.11	NS	0.77 (0.56–1.06)
	GG	104 (0.475)	264 (0.431)	1.24	0.27	NS	1.19 (0.88–1.63)
	A	145 (0.331)	420 (0.343)	0.21	0.68	NS	0.95 (0.75–1.19)
	G	293 (0.669)	804 (0.657)	0.21	0.68	NS	1.06 (0.84–1.33)
rs10893872	CC	82 (0.374)	175 (0.286)	5.91	0.017	NS	1.50 (1.08–2.07)
	CT	103 (0.47)	316 (0.516)	1.37	0.27	NS	0.83 (0.61–1.13)
	TT	34 (0.155)	121 (0.198)	1.91	0.19	NS	0.75 (0.49–1.13)
	C	267 (0.61)	666 (0.544)	5.62	0.018	NS	1.31 (1.05–1.63)
	T	171 (0.39)	558 (0.456)	5.62	0.018	NS	0.76 (0.61–0.96)

P_c_, Bonferroni corrected p value. NS=Not significant. OR=odds ratio. 95% CI=95% confidence interval.

## Discussion

The present study did not show an association between *miR-146a* and *Ets-1* polymorphisms and FUS in a Chinese Han population. A further stratification analysis according to gender or extraocular findings also did not show an association.

Although the etiology and pathogenesis of FUS are not fully understood, viral infections and autoimmunity have been presumed to be involved in its development, as supported by the demonstration of local intraocular antiviral antibody production, increased expression of interferon-gamma (IFN-γ) and interleukin 6 in Fuchs’ patients [23-25]. Viral infection has been shown to be initially recognized by host pattern-recognition receptors (PRRs), including Toll like receptors (TLRs), and has been found to be able to up- or down-regulate the expression of miRNAs in host cells [26]. The role of miR-146a in the innate immune response was identified recently by showing that it could act as a negative feedback regulator in TLR signaling by targeting IL-1R-associated kinase (IRAK) 1 and TNF receptor associated factor 6 (TRAF6) [27]. Other reports further showed that Epstein-Barr virus (EBV)-encoded latent membrane protein 1 and pro-inﬂammatory cytokine IL-1β also induced the expression of miR-146a [16,28,29], miR-146a was decreased by estrogen treatment and in turn led to the augmentation of LPS-induced IFN-γ in splenic lymphocytes [17]. All these studies collectively indicated that miR-146a is indeed involved in the much more complex regulatory network of innate immunity and especially during the viral infection process. In view of these findings, we chose miR-146a and the transcription factor Ets-1 as candidates in an association study of FUS in a Chinese Han population.

Since there are numerous SNPs in a candidate gene and only a few SNPs may be relevant in the pathogenesis of disease, it is extremely important to determine how many and which SNPs should be selected as optimal candidates within the gene to be tested in an association study. In this study, we chose three SNPs of miR-146a and Ets-1 mainly based on earlier studies [22,30,31]. The SNP rs2910164 tested in our study is the only one reported in the miR-146a precursor and has been shown to have a quantitative impact on the total amount of miR-146a [30]. This SNP might reduce the efficiency of processing the pre-miRNA into the mature miRNA [30]. SNP rs2910164 was shown to be associated with papillary thyroid carcinoma in a Caucasian population and with the risk for hepatocellular carcinoma in a Chinese population [30,31]. Our inability to detect an association between rs2910164 and FUS is consistent with earlier findings reported in ulcerative colitis or rheumatoid arthritis in Japanese [32] or Chinese populations [33]. Other two SNP tested in the present study, rs1128334 and rs10893872, are located in the 3′ untranslated region (3′UTR) of *Ets-1*. The post-transcriptional regulatory effect of miRNA on certain subsets of mRNAs (mRNAs) is mediated by binding to their 3′UTR [34]. These two SNPs have been reported to be associated with SLE in an Asian population [22]. Our findings that SNPs rs1128334 and rs10893872 were not associated with FUS may be explained by the fact that the pathways leading to SLE and FUS are different and that other genetically determined risk factors are operational in these diseases.

As a case-control association study may be influenced by various factors, the following efforts were used to validate the results. The healthy controls and patients were strictly age-, sex-, and ethnically matched to exclude the possible influence of stratification of the population. Individuals with ocular inflammation and any autoimmune disease were excluded from the healthy controls by careful inquiry of history and, if necessary, by relevant examinations. When the number of cases is relative small, the ratio of controls to cases can be raised to improve the ability to ﬁnd important variants [35]. Therefore, we have increased the number of controls to improve the power of the study. To validate the result of genotyping by PCR-RFLP, 20% of the total samples were randomly chosen and analyzed by direct sequencing to validate the method employed in this study.

It is worthwhile to point out that there were several limitations in the present study. The sample of the patients in our study seemed to be relatively small and only Han Chinese cohorts were included. Therefore, the results observed in this study need to be confirmed using a large sample size and in other ethnic patients with FUS. In addition, although we failed to find any association of the investigated three SNPs with FUS, our study does not rule out the possibility that other SNPs of *Ets-1* are associated with this disease. More studies are needed to clarify this issue.

In conclusion, our study revealed that the rs2910164, rs1128334, and rs10893872 SNPs were not associated with the susceptibility to FUS in this population. Further studies are necessary to elucidate the association of *miR-146a* and *Ets-1* polymorphisms with FUS using larger samples and in multiethnic populations.
